# The clinical significance of serum chitinase 3‐like 1 in hepatitis B–related chronic liver diseases

**DOI:** 10.1002/jcla.23200

**Published:** 2020-01-08

**Authors:** Zhenluo Jiang, Shuwei Wang, Jiancheng Jin, Sheng Ying, Zhigang Chen, Dedong Zhu, Bingxiu Xiao, Yaoren Hu, Yunsong Qian, Ting Cai, Liyun Fu

**Affiliations:** ^1^ Department of Hepatology HwaMei Hospital University Of Chinese Academy Of Sciences Ningbo China; ^2^ Department of Biochemistry and Molecular Biology Zhejiang Key Laboratory of Pathophysiology Medical School of Ningbo University Ningbo China; ^3^ Key Laboratory of Diagnosis and Treatment of Digestive System tumors of Zhejiang Province Ningbo China

**Keywords:** CHI3L1, diagnosis, hepatitis B, hepatocellular carcinoma, liver fibrosis

## Abstract

**Aim:**

In the present study, we purposed to determine serum chitinase 3‐like 1 (CHI3L1) expression characteristics in chronic liver diseases monoinfected with hepatitis B virus and analyze its diagnostic value in liver fibrosis.

**Methods:**

A total of 467 chronic hepatitis B (CHB) patients, 312 liver cirrhosis (LC) patients, and 104 hepatocellular carcinoma (HCC) patients at our institution were enrolled, and clinical indicators were analyzed.

**Results:**

Our data have shown that the expression level of serum CHI3L1 was steadily increased from CHB to LC to HCC (*P* < .001). Serum CHI3L1 expression levels were positively associated with liver stiffness measurement (LSM), fibrosis‐4 (FIB‐4) index, aspartate aminotransferase‐to‐platelet ratio index (APRI), and HCC stage. The receiver operating characteristic (ROC) curve proved that serum CHI3L1 was superior to other noninvasive methods (LSM, FIB‐4, and APRI) with an area under the ROC curve (AUC) of 0.97 in diagnosing significant fibrosis.

**Conclusions:**

Serum CHI3L1 harbors significant clinical value in chronic liver diseases infected with hepatitis B virus, especially in the diagnosis of fibrosis.

## 1 INTRODUCTION

With an estimated prevalence of more than 240 million worldwide, hepatitis B virus (HBV) infection is a major public healthcare burden, especially in Asia. Chronic hepatitis B (CHB) is one of the most reasons of liver cirrhosis (LC) and hepatocellular carcinoma (HCC).^1^, ^2^ Hepatic fibrosis is a repairing response of hepatocytes to chronic damage caused by toxic damage, viral infections, autoimmune conditions, and metabolic and genetic diseases.^3^ Long‐term progression of liver fibrosis can lead to LC and HCC.^4^ It is well known that LC of any cause is the major risk factor of HCC.^5^ According to the guideline of American Association for the Study of Liver Diseases (AASLD) and the Guideline of Prevention and Treatment for CHB from Chinese Society of Hepatology, Chinese Medical Association, proper assessment of liver fibrosis stages is vital to determining the timing of antiviral therapy.^6^ Hence, it is very considerable for us to detect the degree of liver fibrosis correctly.

In the past few years, liver biopsy is the gold standard for evaluating the stage of liver fibrosis.^7^ Nevertheless, for some reasons, such as invasiveness, poor consistency, high cost, or serious complications, its application is greatly limited, especially in dynamic monitoring disease evolution.^8^ In recent years, some noninvasive detection techniques have emerged, such as transient elastography (TE), FibroTest, and ActiTest. However, their accuracy is far from satisfactory.^9^ Hence, there is an unmet need to discover novel potential biomarkers to improve the accuracy of fibrosis detection.

Chitinase 3‐like 1 (CHI3L1) is a member of the chitinase family, which can combine with chitin but lacks chitinolytic activity.^10^ CHI3L1 makes a difference in the process of inflammation and tissue remodeling^11^ Recently, CHI3L1 mRNA expression was characterized by tissue specificity, which level was highest in the liver.^12^ Johansen et al^13^ reported that the expression level of CHI3L1 in serum could evaluate the degree of liver fibrosis. These proofs suggest that CHI3L1 can be promising novel targets for fibrosis diagnosis.

The purposes of this study were to explore the expression feature of serum CHI3L1 in the different stages of patients infected with HBV and to analyze the diagnostic value of CHI3Ll in liver fibrosis combined with the pathological data of liver biopsy. Our results indicate that CHI3L1 could serve as a serum biomarker not only for diagnosing liver fibrosis but also for monitoring the process of chronic liver diseases monoinfected with HBV.

## 2 METHODS

### 2.1 Patients and ethics statement

We collected serum samples from patients with chronic liver diseases monoinfected with HBV who were treated at HwaMei Hospital, University Of Chinese Academy Of Sciences (Ningbo No.2 hospital) from December 2018 to July 2019. Among them, 467 were patients with CHB (patients with other liver diseases were excluded), 312 were patients with LC related to CHB (patients with other serious systemic diseases were excluded), and 104 were patients with HCC related to CHB (patients who received prior treatment of their tumor or had history of other tumors were excluded). Diagnostic criteria for CHB and LC were based on the Guideline of Prevention and Treatment for Chronic Hepatitis B (2nd version).^1^ Diagnostic criteria for HCC were based on the Guidelines for Diagnosis and Treatment of Primary Liver Cancer in China (2017 Edition).^14^ All aspects of this study were approved by the Human Research Ethics Committee from HwaMei Hospital, University Of Chinese Academy Of Sciences. Clinical information was obtained under an institutional review board approved study protocol, and written informed consent was signed from each patient.

### 2.2 Detection of clinical indicators

All clinical indicators were tested during the same period by standard methods in HwaMei Hospital, University Of Chinese Academy Of Sciences, including blood platelet count, alanine aminotransferase (ALT), aspartate aminotransferase (AST), albumin, bilirubin, prothrombin time (PT), α‐fetoprotein (AFP), and liver stiffness measurement (LSM). Blood platelet count was measured by ADVIA 2120i (Siemens). Liver function was measured by ADVIA XPT (Siemens). PT was measured by INNOVANCE PFA‐200 (Siemens). AFP was measured by ADVIA^®^ Centaur XPT (Siemens). LSM was assessed by two experienced investigators in close cooperation through Fibro Scan (Echosens), without knowledge of clinical information and biochemical indicators.

### 2.3 Serum CHI3L1 protein level determination

The levels of CHI3L1 in the serum were measured by CHI3L1 ELISA Kit manufactured by Proprium Biotech Co.

### 2.4 Histological assessment

Among the patients with CHB, 50 cases have received ultrasound‐guided liver biopsy. METAVIR scoring system was used to assess the fibrosis stage (F0 = no fibrosis, F1 = portal fibrosis without septa, F2 = portal fibrosis with rare septa, F3 = numerous septa without cirrhosis, F4 = cirrhosis) by two experienced pathologists in blind manner.^15^

### 2.5 Clinical score evaluation

The severity of liver dysfunction was evaluated by Child‐Pugh score in the LC group.^16^ HCC staging was based on the tumor‐node‐metastasis (TNM) staging system^17^ and the Barcelona Clinic Liver Cancer staging system (BCLC).^18^ Noninvasive fibrosis scores were evaluated by aspartate aminotransferase‐to‐platelet ratio index (APRI) and fibrosis‐4 (FIB‐4) index. APRI = [(AST(U/L)/upper limit of normal range (ULN))/platelet(10^9^/L)]×100^19^, FIB4 = (age(years) × AST(U/L))/[platelet(10^9^/L) × ALT(U/L)^1/2^].^20^

### 2.6 Statistical analysis

All statistical analyses were done by GraphPad Prism 7.0 (GraphPad Software), the Statistical Product and Service Solutions (SPSS) 18.0 software package (IBM), and MedCalc software (MedCalc software bvba). In this study, Kruskal‐Wallis test, chi‐square test, Mann‐Whitney *U* test, and Pearson's correlation coefficient test were used. The diagnostic power was obtained by using receiver operating characteristic (ROC) curve. *P* value of .05 or less was deemed statistically significant.

## 3 RESULTS

### 3.1 Characteristics of enrolled patients

The clinical characteristics of all enrolled patients are shown in Table 1. The characteristics of CHB patients who have received ultrasound‐guided liver biopsy are shown in Table 2.

**Table 1:** The clinical characteristics of enrolled patients (data are mean ± SD)

Parameter	CHB	LC	HCC
Number	467	312	104
Gender(M/F)	314/153	222/90	91/13
Age(years)	42.27 ± 9.46	50.62 ± 10.88	59.74 ± 10.76
TBil (μmol/L)	14.59 ± 17.16	27.48 ± 48.86	32.94 ± 53.36
ALT (U/mL)	66.99 ± 175.09	62.23 ± 139.73	50.03 ± 54.22
AST (U/mL)	48.47 ± 133.14	53.64 ± 102.50	74.83 ± 77.35
Albumin (g/L)	44.19 ± 3.58	40.35 ± 7.08	35.30 ± 6.66
PT (s)	NA	13.98 ± 4.38	14.64 ± 3.05
CH3L1(ng/mL)	81.11 ± 86.17	141.5 ± 140.89	245.9 ± 189.55
AFP(ng/mL) (n)			
>20			48
≤20			49
Ascites (n)			
No		261	
Mild		37	
Moderate and severe		14	
HE stage(n)			
0		308	
1+2		1	
3+4		3	
Child‐Pugh stage(n)			
A		247	
B		45	
C		20	
TNM stage(n)			
Ⅰ+Ⅱ			56
Ⅲ+Ⅳ			48
BCLC stage(n)			
A+B			64
C+D			40

**Table 2:** Characteristics of CHB patients who have been carried out ultrasound‐guided liver biopsy (data are mean ± SD)

Parameter	F0‐F1	F2‐F3
Number	29	21
Gender(M/F)	15/14	11/10
Age(years)	39.83 ± 9.75	42.05 ± 9.35
TBil(μmol/L)	12.73 ± 7.25	12.93 ± 4.18
ALT(U/mL)	29.59 ± 15.23	40.19 ± 51.98
AST(U/mL)	29.17 ± 31.61	31.33 ± 25.80
Albumin(g/L)	43.39 ± 3.88	42.05 ± 3.38
Platelet(×10^9^/L)	206.83 ± 43.22	158.86 ± 40.95
CH3L1(ng/mL)	50.09 ± 16.28	131.03 ± 102.46
LSM (Kpa)	5.36 ± 1.37	10.05 ± 5.35

### 3.2 Different serum levels of CHI3L1 in CHB, LC, and HCC

**Figure 1:**
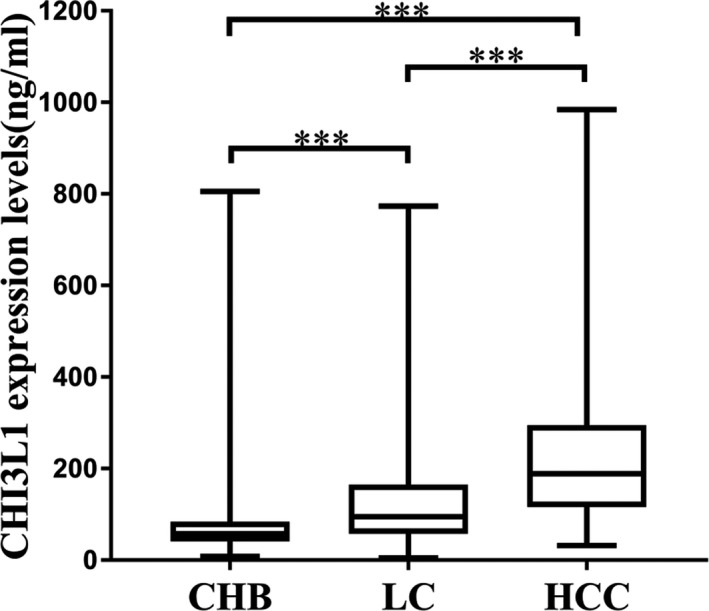
CHI3L1 expression features in the cohort of chronic hepatitis B (CHB) (n = 467) (81.11 ± 86.17 ng/mL), liver cirrhosis (LC) (n = 312) (141.5 ± 140.89 ng/mL), and hepatocellular carcinoma (HCC) (n = 104) (245.9 ± 189.55 ng/mL). Data are means ± SD. ***P<.001

As shown in Figure 1, the expression levels of CHI3L1 were the highest in the HCC group, the lowest in the CHB group, and the middle in the LC group.

### 3.3 CHI3L1 expression levels were correlated with Child‐Pugh stage in LC patients

**Figure 2:**
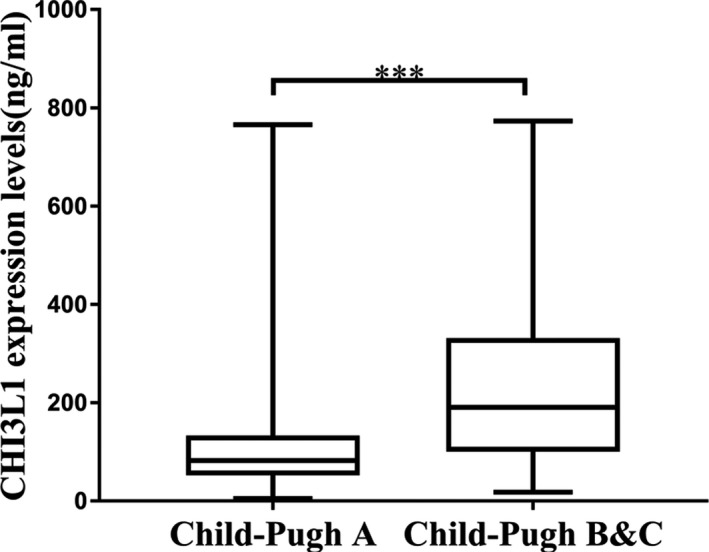
Different expression of CHI3L1 in LC patients according to Child‐Pugh classification. Child‐Pugh A (n = 247): 115.21 ± 113.81 ng/mL; Child‐Pugh B (n = 45) &C (n = 20): 241.58 ± 183.95 ng/mL. Data are means ± SD. ***P<.001

We grouped the LC patients according to Child‐Pugh score. Among them, 247 cases in Child‐Pugh A group, 45 cases in Child‐Pugh B group, and 20 cases in Child‐Pugh C group. Patients were then divided into good liver function group (Child‐Pugh A) and liver dysfunction group (Child‐Pugh B and Child‐Pugh C). The expression levels of CHI3L1 in good liver function group were lower than those in liver dysfunction group (Figure 2, *P* < .001).

### 3.4 CHI3L1 expression levels were correlated with TNM stage, BCLC stage, and AFP in HCC patients

**Figure 3:**
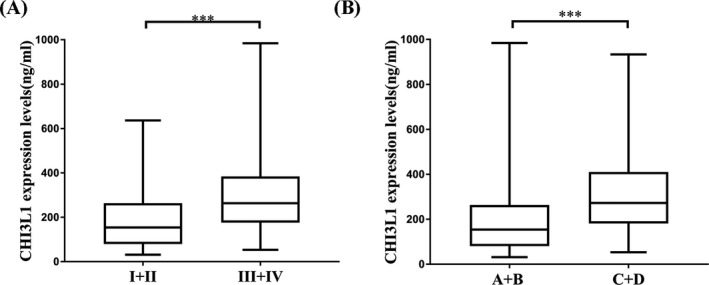
CHI3L1 expression levels were correlated with HCC stages. A, Increased expression levels of CHI3L1 from TNM stage I + II (n = 56) (193.52 ± 154.72 ng/mL) to III + IV (n = 48) (307.09 ± 208.89 ng/mL). B, Increased expression levels of CHI3L1 from BCLC stage A + B (n = 64) (205.27 ± 178.96 ng/mL) to C + D (n = 40) (311.00 ± 190.01 ng/mL). ***P<.001

**Figure 4:**
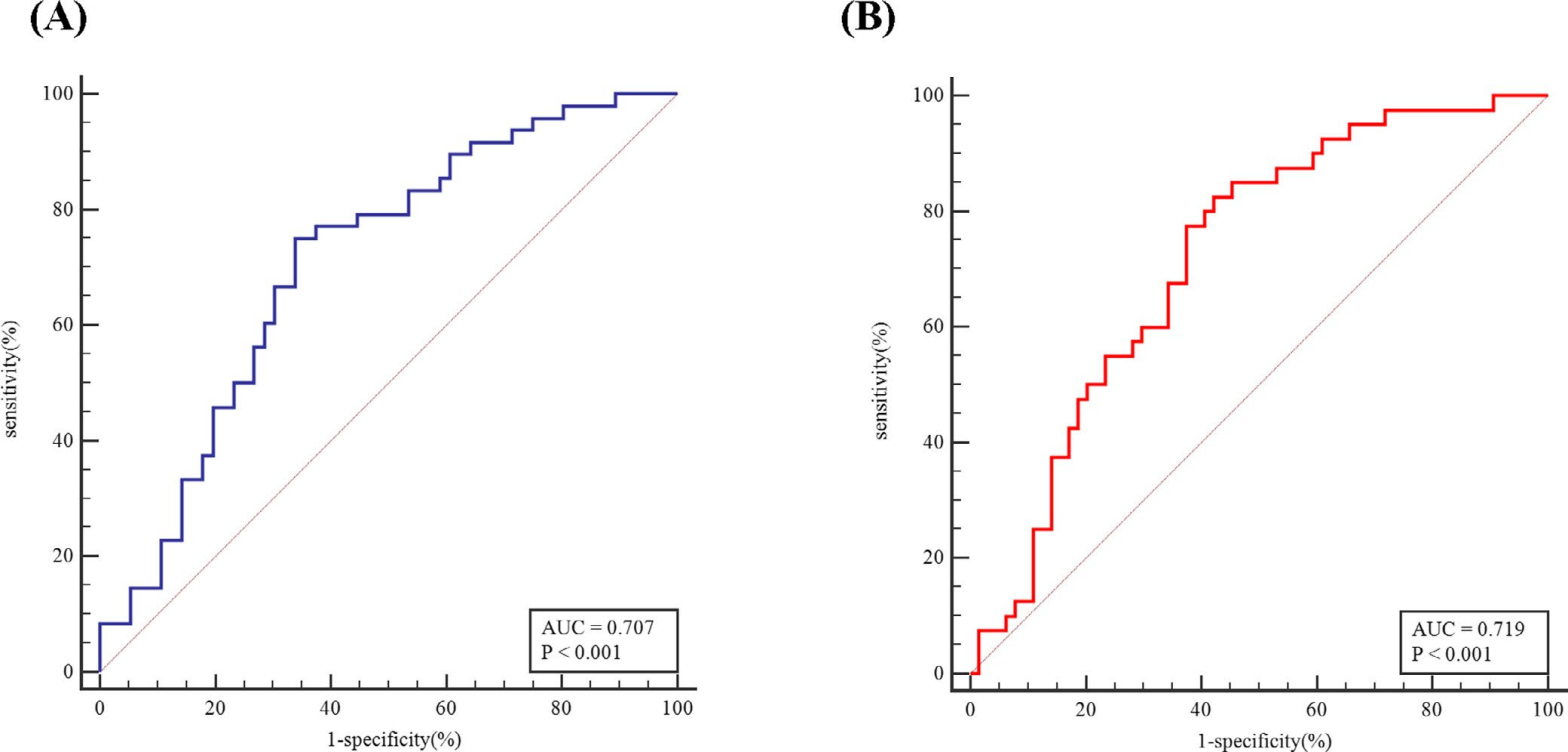
Diagnostic value of CH3L1 for HCC staging. A, Distinguishing TNM stage Ⅰ+Ⅱ from Ⅲ+Ⅳ. B, Distinguish BCLC stage A + B from BCLC C + D

**Figure 5:**
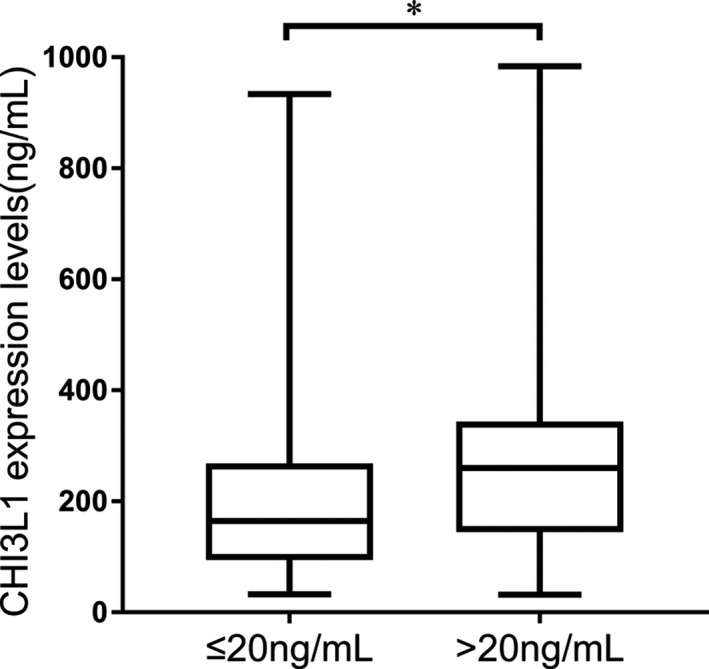
CHI3L1 expression levels were correlated with serum AFP levels. AFP ≤ 20 ng/mL (n = 49): 216.36 ± 180.76 ng/mL; AFP > 20 ng/mL (n = 48):283.29 ± 199.37 ng/mL. Data are means ± SD. *P<.05

According to TNM stage or BCLC stage, HCC patients were divided into two groups; details are shown in Table 1. As shown in Figure 3, CHI3L1 expression levels were related to TNM stage (*P* < .001) and BCLC stage (*P* < .001). ROC curves were then constructed to assess the diagnostic significance of CHI3L1 in HCC staging. 180.23 ng/mL of CHI3L1 was taken as the cut‐off point to distinguish TNM stageⅠ+Ⅱ from stage Ⅲ+Ⅳ, AUC = 0.707, specificity = 0.661, sensitivity = 0.750; taking 168.59 ng/mL of CHI3L1 as the cut‐off point to distinguish BCLC stage A + B from stage C + D, AUC = 0.707, specificity = 0.661, sensitivity = 0.750 (Figure 4). Ninety‐seven patients have been tested for AFP in the HCC group. According to a threshold of 20 ng/mL, HCC patients were divided into two groups; details are shown in Table 1. As shown in Figure 5, CHI3L1 expression levels were related to serum AFP levels (*P* < .05).

### 3.5 CHI3L1 expression levels were correlated with LSM, APRI index, and FIB‐4 index in CHB patients

**Figure 6:**
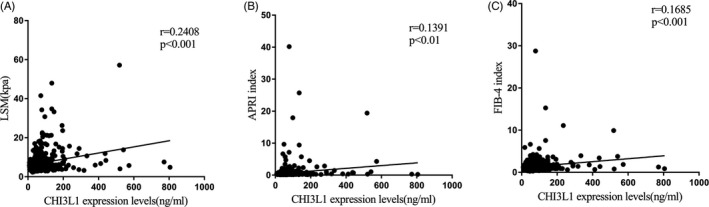
Relationships between serum CHI3L1 levels and LSM, APRI index, and FIB‐4 index in CHB patients. A, CHI3L1 levels had positive correlations with LSM (*r* = .241, ***P < .001). B, CHI3L1 levels had positive correlations with APRI index (*r* = .139, **P < .01). C, CHI3L1 levels had positive correlations with FIB‐4 index (*r* = .162, ***P < .001)

The correlation analysis shown positive correlations between serum CHI3L1 levels and LSM (r = 0.2408, *P* < .001) (Figure 6A), between serum CHI3L1 levels and APRI index (*r* = .1391, *P* < .01) (Figure 6B), and between serum CHI3L1 levels and FIB‐4 index (*r* = .1685, *P* < .001) (Figure 6C).

### 3.6 Potential value of CHI3L1, LSM, APRI index, and FIB‐4 index as biomarkers for fibrosis diagnosis

**Figure 7:**
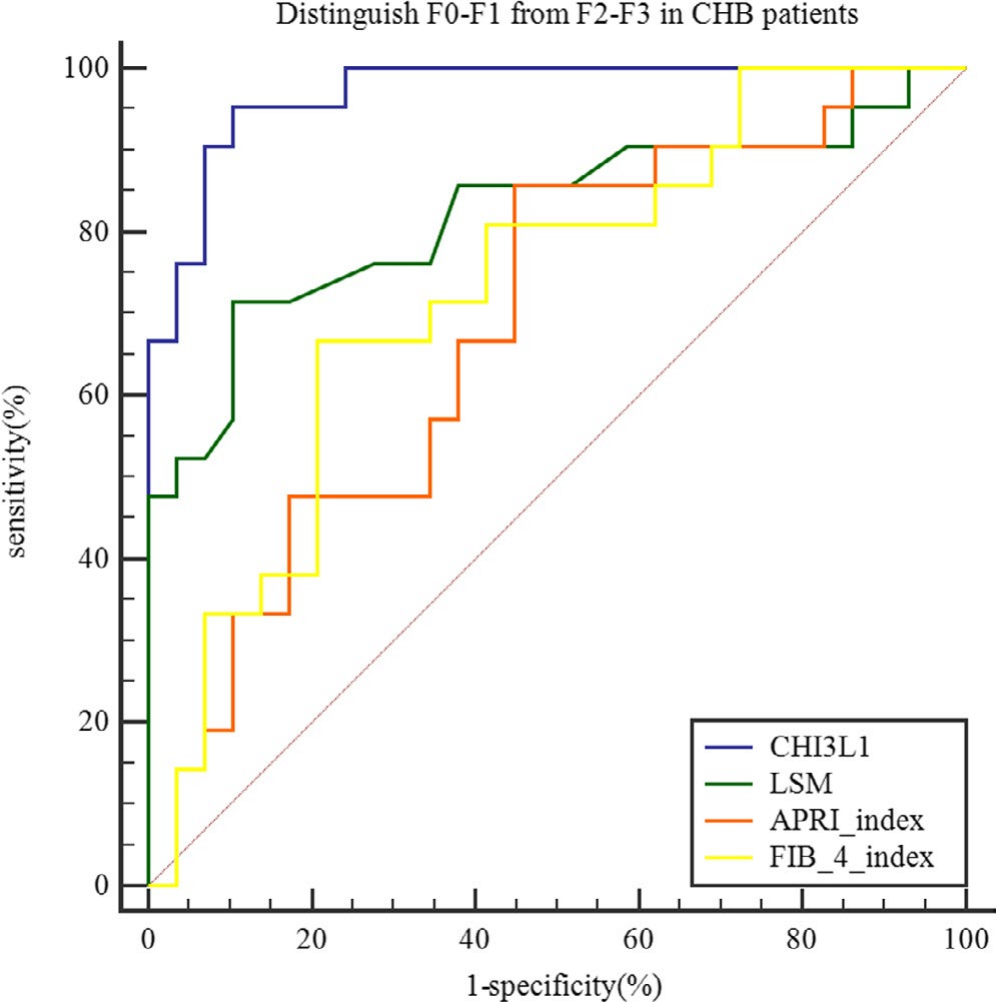
Diagnostic value of CHI3L1, LSM, APRI index, and FIB‐4 index for distinguishing F0‐F1 (n = 29) (50.09 ± 16.28 ng/mL) from F2‐F3 (n = 21) (131.03 ± 102.46 ng/mL) in CHB patients

There were 50 CHB patients on whom ultrasound‐guided liver biopsy has been carried out. Among them, 1 patient was stage F0, 28 patients were stage F1, 8 patients were stage F2, and 13 patients were stage F3. ROC curves were then constructed to assess the diagnostic value of CHI3L1, LSM, APRI index, and FIB‐4 index in fibrosis diagnosis. The diagnostic power of CHI3L1 (AUC = 0.970, specificity = 0.897, sensitivity = 0.952, cut‐off value = 68.75) outperforms LSM (AUC = 0.823, specificity = 0.897, sensitivity = 0.714, cut‐off value = 6.7), APRI index (AUC = 0.688, specificity = 0.552, sensitivity = 0.857, cut‐off value = 0.289), and FIB‐4 index (AUC = 0.729, specificity = 0.793, sensitivity = 0.667, cut‐off value = 1.175) in distinguishing F0‐F1 cases from F2‐F3 cases (Table 3, Figure 7).

**Table 3:** Diagnostic values in distinguishing F0‐F1 cases from F2‐F3 cases

Markers	AUC	Specificity	Sensitivity	Cut‐off value
CHI3L1	0.970	0.897	0.952	68.750
LSM	0.823	0.897	0.714	6.700
APRI index	0.688	0.552	0.857	0.289
FIB‐4 index	0.729	0.793	0.667	1.175

## 4 DISCUSSION

The gene of CHI3L1 is located on human chromosome 1q32.1, which is composed of 10 exons. CHI3L1 belongs to the glycoside hydrolase 18 family of chitinases and is secreted by activated macrophages, chondrocytes, neutrophils, and synovial cells.^21^ Nowadays, there are a few of studies on the association of CHI3L1 with liver diseases. For example, Higashiyama et al^22^ reported that CHI3L1 promotes liver fibrosis by inhibiting apoptosis in hepatic macrophages. In hepatitis C‐infected population, Kamal et al^23^ discovered that CHI3L1 can accurately determine the speed of fibrosis progression, identifying whether the patient is in the stage of rapid fibrosis or stable disease. Huang et al^8^ identified CHI3L1 could be a good marker of substantial fibrosis with high degree of accuracy, specificity, and sensitivity. Wang et al^24^ demonstrated that CHI3L1 is not only a noninvasive marker for assessing liver fibrosis before treatment in CHB patients, but also a potential marker for monitoring liver fibrosis changes during treatment.

In this study, serum levels of CHI3L1 were measured in CHB patients, LC patients, and HCC patients and we studied the stage expression characteristics in different stages of chronic liver diseases related to hepatitis B. As Figure 1 shows, the expression levels of CHI3L1 were increased successively from CH to LC to HCC. The result suggested that CHI3L1 may have clinical value in assessing different stage of chronic liver diseases and could be a disease evolution monitoring indicator.

Child‐Pugh classification is widely used to determine liver function in LC patients according to five parameters: bilirubin, serum albumin concentrations, prothrombin time (international normalized ratio), the presence and severity of ascites, and hepatic encephalopathy (HE), and is helpful in guiding treatment, judging prognosis, and assessing drug efficacy.^25^ Our research has manifested that the levels of CHI3L1 in the group with poor liver function (Child‐Pugh B and Child‐Pugh C) were higher than those in the group with good liver function (Child‐Pugh A) (Figure 2), indicating that CHI3L1 as a quantitative index can directly and effectively evaluate the levels of liver function in LC patients. In our recruited HCC group, the percentage of patients who have LC background was approximately 75.0% (78/104). The liver function (TBil, ALT, AST, albumin) and PT of patients in the LC group were better than those in the HCC group (*P* < .001). This suggested that the different expression levels of CHI3L1 between HCC group and LC group may be caused by differences in liver function. The underlying mechanism requires further research.

Precision staging of HCC patients is critical for determining treatment options and prognosis.^26^ As Figure 3 shows, the serum levels of CHI3L1 in HCC patients with early or moderate stages were lower than those with advanced stages. This study indicated that CHI3L1 could be a potential noninvasive surrogate biomarker for HCC staging. AFP was the most widely used biomarker in HCC for decades.^27^ As Figure 5 shows, the serum levels of CHI3L1 in HCC patients with low AFP levels were lower than those with high AFP levels. This suggested that CHI3L1 may have potential relationship with AFP, which needs further research.

In recent years, there are some noninvasive methods for fibrosis diagnosing in liver, such as TE, APRI index, and FIB‐4 index. Although TE has many advantages, including simple operation, good repeatability, and high accuracy, successful measurement of TE is restricted by size of the intercostal space, obesity, experience of operators, and the value of LSM is affected by cholestasis and fatty degeneration, hepatic inflammation, among other factors.^28^ APRI index and FIB‐4 index are ground on clinical and biochemical parameters.^29^ Patients can benefit from these two indices, because of their low price, good repeatability, and ease of acceptance.^30^ However, the sensitivity and accuracy of these two indices in diagnosing CHB‐related fibrosis are not very satisfactory.^31^ Correlation analysis in this study revealed that CHI3L1 expression levels were positively related to LSM, APRI index, and FIB‐4 index in CHB patients (Figure 6). According to current guidelines, patients with significant fibrosis (≥F2) are recommended for antiviral therapy.^32^ Thus, patients who underwent liver biopsy were divided into two groups, one with mild fibrosis (F0, F1) and another with moderate and significant fibrosis (F2, F3). ROC analysis indicated that CHI3L1 had the best diagnostic performance among the noninvasive methods tested for differentiating these two groups (Figure 7).

In summary, we found that serum CHI3L1 has stage expression feature in different stage liver disease with chronic HBV infection. Despite these, our results indicated that CHI3L1 might be utilized as novel biomarker for evaluating the different levels of liver function and HCC staging. Further research demonstrated that CHI3L1 had positive relationship with noninvasive fibrosis diagnosis methods (LSM, APRI index, FIB‐4 index) and could be a feasible marker for diagnosing significant fibrosis with a high degree of accuracy, specificity, and sensitivity. But, there are some limitations in this study. Firstly, the number of CHB patients on whom liver biopsy has been carried out is small; secondly, this is single‐center study, and multicenter study is to be encouraged to evaluate external performance; thirdly, the exact mechanism of CHI3L1 in hepatocarcinogenesis needs to be further investigated; and fourthly, the prognostic value of CHI3L1 in liver disease is not discussed in this study.
